# Hemostasis of active bleeding in the duodenal bulb using a sharp-clawed reopenable endoclip under gel immersion endoscopy

**DOI:** 10.1055/a-2512-0792

**Published:** 2025-02-05

**Authors:** Daiki Kitagawa, Shusei Fukunaga, Yumie Kobayashi, Akinobu Nakata, Mitsuhiro Kono, Masaki Ominami, Yasuhiro Fujiwara

**Affiliations:** 1 Department of Gastroenterology, Osaka Metropolitan University Graduate School of Medicine, Osaka, Japan

An 87-year-old woman who was taking nonsteroidal anti-inflammatory drugs for fracture of the right distal radius underwent esophagogastroduodenoscopy (EGD) for melena at another hospital. The EGD revealed a large ulcer with a blood clot on the anterior wall of the duodenal bulb ( Fig. 1 ), and she was referred to our hospital for treatment.

**Fig. 1:**
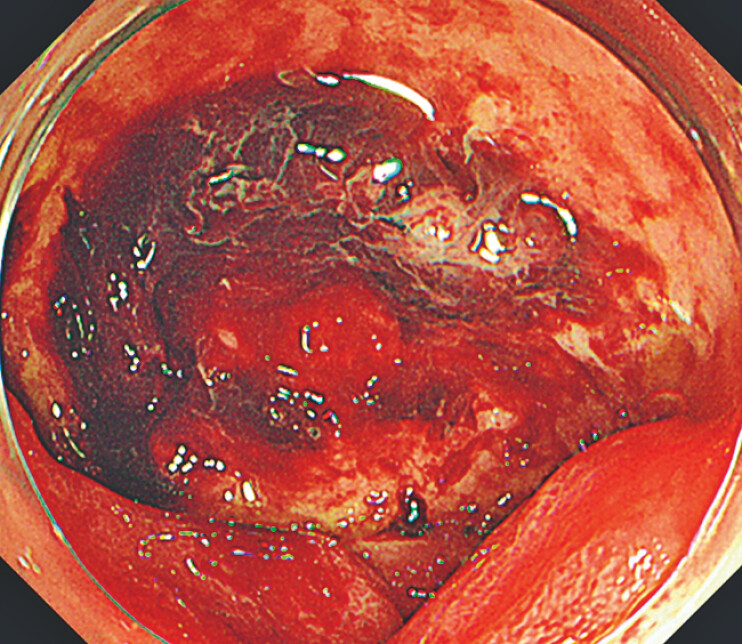
A large ulcer with a blood clot on the anterior wall of the duodenal bulb.

Active bleeding occurred during blood clot removal, but hemostasis by cauterization with hemostatic forceps was difficult ( Video 1 ). The bleeding temporarily ceased due to reduced blood pressure. A conventional reopenable endoclip was used to prevent rebleeding. However, the endoclip could not completely capture the large exposed vessel, leading to rebleeding and, ultimately, dislocation of the clip. While the bleeding point could not be visualized under carbon dioxide insufflation, it was visualized with red dichromatic imaging using the gel immersion technique 1 ( Fig. 2 ). As additional cauterization may result in delayed perforation, a reopenable endoclip with a sharp claw (Mantis Clip; Boston Scientific, Marlborough, Massachusetts, USA) 2 3 , which was able to grasp the vessel along with the surrounding tissue ( Fig. 3 ), was applied, and hemostasis was achieved ( Fig. 4 ).

Hemostasis of active bleeding in the duodenal bulb using a sharp-clawed reopenable endoclip under gel immersion endoscopy.Video 1

**Fig. 2:**
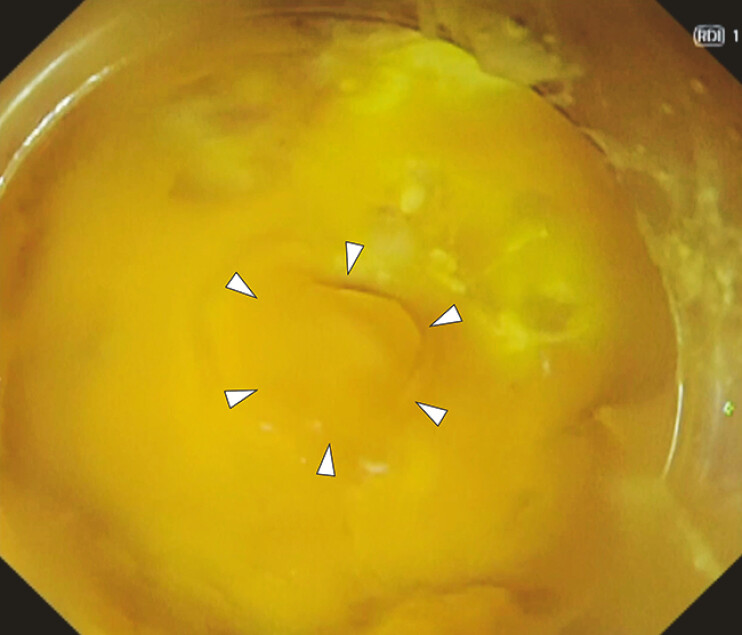
The bleeding point was visualized by gel immersion technique (arrowheads).

**Fig. 3:**
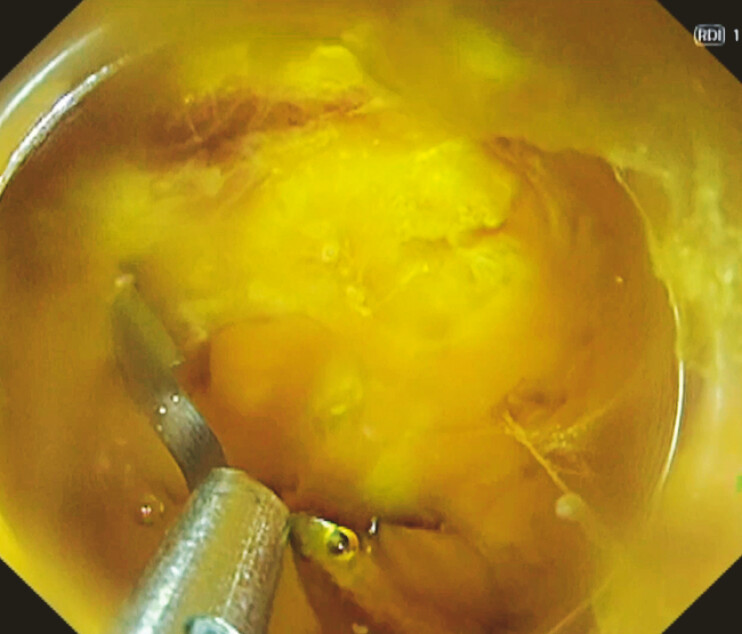
Use of a reopenable endoclip with a sharp claw (Mantis Clip; Boston Scientific, Marlborough, Massachusetts, USA) for the visible bleeding point.

**Fig. 4:**
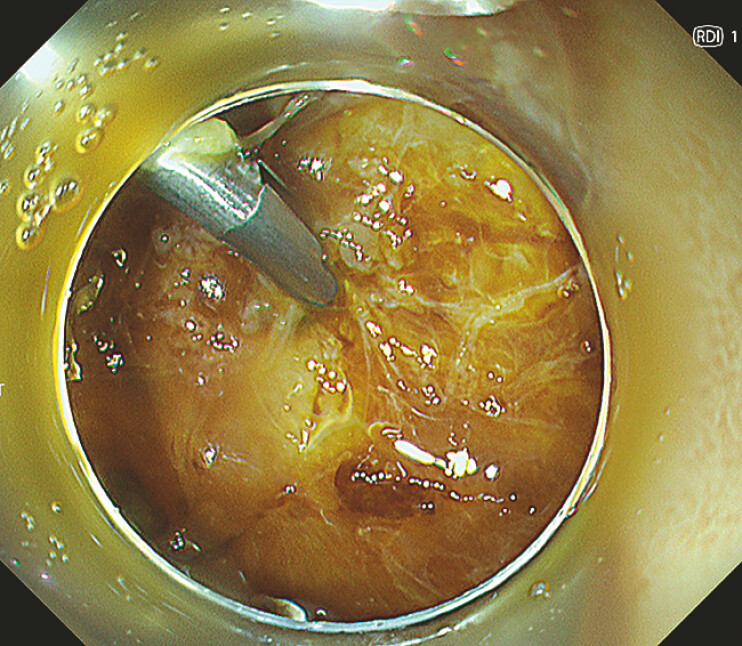
Hemostasis was achieved using the endoclip.

A follow-up endoscopy at 4 days postoperatively revealed loss of the endoclip, but the ulcer was healing ( Fig. 5 ). Oral intake was resumed 7 days postoperatively, and the patient was transferred to another hospital for walking rehabilitation 17 days postoperatively.

**Fig. 5:**
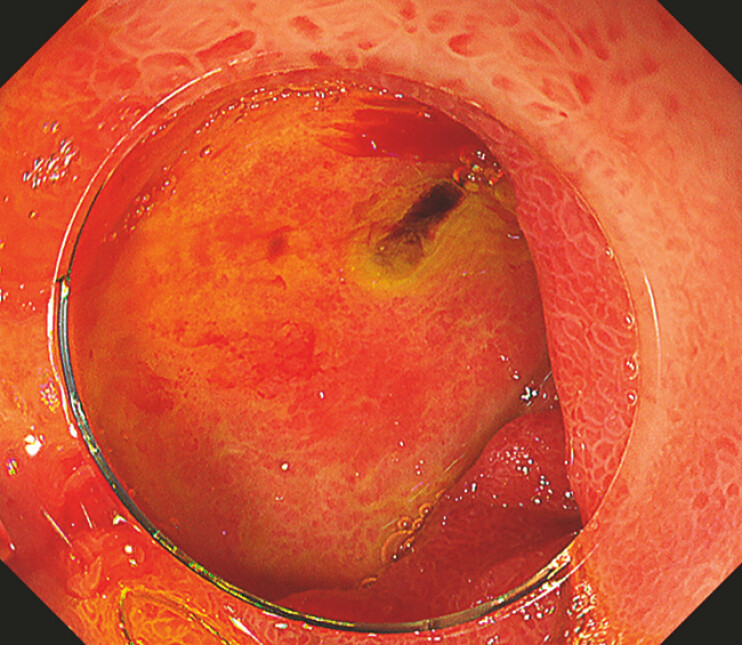
The ulcer was healing 4 days after the procedure.

Hemorrhagic duodenal ulcers are a life-threating disease 4 . In cases of endoscopic hemostasis failure, transcatheter arterial embolization or surgery are necessary 5 . Endoscopic hemostasis is often difficult because the bleeding point is not visible, and the large vessel in the ulcer cannot be completely captured. In the current case, these issues were solved using the gel immersion technique and endoclipping with a sharp claw, leading to the clinical advantage of avoiding invasive transcatheter arterial embolization or surgery.

Endoscopy_UCTN_Code_TTT_1AO_2AD

## Notes

Endoscopy E-Videos https://eref.thieme.de/e-videos
*E-Videos* is an open access online section of the journal *Endoscopy* , reporting on interesting cases and new techniques in gastroenterological endoscopy. All papers include a high-quality video and are published with a Creative Commons CC-BY license. Endoscopy E-Videos qualify for HINARI discounts and waivers and eligibility is automatically checked during the submission process. We grant 100% waivers to articles whose corresponding authors are based in Group A countries and 50% waivers to those who are based in Group B countries as classified by Research4Life (see: https://www.research4life.org/access/eligibility/ ). This section has its own submission website at https://mc.manuscriptcentral.com/e-videos .
